# Bioactive Peptide Fractions from Mung Bean Protein Hydrolysate: Antioxidant Activity, Anti-Inflammatory Effects, and Immunomodulatory Responses in RAW 264.7 Macrophages

**DOI:** 10.3390/ijms27156882

**Published:** 2026-08-01

**Authors:** Worrapob Chaisan, Kanokwan Promjeen, Komgrit Eawsakul, Chawan Manaspon, Niramon Utama-ang

**Affiliations:** 1 Cluster of High Value Products from Thai Rice and Plants for Health, Chiang Mai University, Chiang Mai 50100, Thailand; worrapob.ch@gmail.com; 2 Division of Product Development Technology, Faculty of Agro Industry, Chiang Mai University, Chiang Mai 50100, Thailand; kanokwan_pr@cmu.ac.th; 3 Applied Thai Traditional Medicine, School of Medicine, Walailak University, Nakhon Si Thammarat 80160, Thailand; komgrit.ea@wu.ac.th; 4 Biomedical Engineering Institute, Chiang Mai University, Chiang Mai 50100, Thailand

**Keywords:** *Vigna radiata* L., plant-derived peptides, size-based fractionation, oxidative stress, enzyme inhibition, immunomodulation

## Abstract

Amid growing concerns over climate change and the need for sustainable agriculture, the study investigated the antioxidant, anti-inflammatory, and immunomodulatory activities of MBPH fractions. MBPH was produced via alcalase hydrolysis and fractionated by ultrafiltration into >5, 3–5, 1–3, and <1 kDa fractions. Bioactivity exhibited a clear inverse correlation with molecular weight. The <1 and 1–3 kDa fractions demonstrated their highest antioxidant capabilities through their performance in ORAC and ABTS tests, which produced results of 836.51 ± 11.81 µmol TE/g and 19.36 ± 0.71 µmol TE/g, respectively. Both fractions also demonstrated significant in vitro anti-inflammatory potential, with the <1 kDa fraction showing superior activity by inhibiting LOX activity (76.93 ± 1.53%) and heat-induced albumin denaturation (81.82 ± 2.72%). All fractions were non-cytotoxic, preserving >90% cell viability in RAW 264.7 macrophages at 100 µg/mL. The 1–3 kDa MBPH fraction exhibited distinct context-dependent immunomodulatory activity in RAW 264.7 macrophages. At 3 h, the fraction markedly suppressed *Tnf-α* and *Il-1β* expression under LPS stimulation, indicating strong early-stage anti-inflammatory effects. At 6 h, IL-6 secretion further confirmed this dual behavior, with a mild increase under basal conditions (33.15–36.42 pg/mL) and a reduced level under inflammatory conditions (28.25–28.81 pg/mL). This represents an approximate 15–22% lower IL-6 production technology under LPS stimulation compared to basal conditions at equivalent concentrations. The results demonstrate that low-molecular-weight MBPH peptides, particularly the 1–3 kDa fraction, exhibit dual functionality by providing robust antioxidant protection while dynamically rebalancing immune homeostasis, highlighting their immense potential as bioactive ingredients for functional foods of agroindustry.

## 1. Introduction

Non-communicable diseases (NCDs) remain the leading cause of global mortality. Environmental factors, particularly air pollution (e.g., fine particulate matter or PM2.5), significantly exacerbate this burden by inducing oxidative stress and chronic inflammation, often via macrophage overactivation [1,2]. This inflammation-driven pathogenesis underscores the urgent need for safe, food-derived bioactive interventions capable of mitigating such detrimental immune responses.

Plant-derived bioactive peptides have emerged as promising natural immunomodulatory and antioxidant agents. These short protein sequences (typically <50 amino acids) are encrypted within parent proteins and can be released through selective enzymatic hydrolysis using commercial proteases, such as Alcalase and Flavourzyme [3,4]. Mung bean (*Vigna radiata* L.), a widely cultivated leguminous crop, serves as an excellent substrate for this purpose due to its high protein content (20–33%), high digestibility, and rich essential amino acid profile [5,6,7]. Mung bean-derived peptides have demonstrated diverse health-promoting properties, including antioxidant, anti-inflammatory, and antihypertensive activities [8,9,10]. Mechanistically, these peptides exert immunoregulatory effects by modulating key signaling pathways (e.g., NF-κB, MAPK, and Toll-like receptors), thereby downregulating pro-inflammatory cytokines (*Tnf-α*, *Il-6*, *Il-1β*) and restoring immune homeostasis [11,12,13,14,15].

The bioefficacy of these peptides is intrinsically governed by their structural characteristics, notably their molecular size and the presence of hydrophobic amino acids [16]. Low-molecular-weight (LMW) peptides, particularly those below 1 kDa, often exhibit superior antioxidant and antibacterial activities due to enhanced bioavailability and target interactions [17]. Consequently, selective size-based fractionation, such as membrane ultrafiltration, is crucial for reducing matrix complexity, elucidating structure–activity relationships, and isolating highly potent fractions [18,19].

However, although the health-promoting properties of MBPH have been widely reported, most previous studies have focused on crude hydrolysates or have evaluated only individual biological activities. Comprehensive studies systematically integrating molecular weight fractionation with antioxidant activity, cell-free anti-inflammatory activity, cytotoxicity, and macrophage immunomodulatory responses remain limited. Furthermore, the relationship between peptide molecular weight and context-dependent immunomodulatory activity under both basal and inflammatory conditions has not been comprehensively established. Consequently, the molecular weight fraction providing the optimal balance of these complementary biological activities has yet to be identified, limiting the rational development of MBPH as a functional food ingredient.

Addressing this gap, in the present study, the primary aim was to investigate the antioxidant and in vitro anti-inflammatory potentials of MBPH and its fractionated peptides. Furthermore, the immunomodulatory activity of these fractions in RAW 264.7 macrophages was evaluated in environments with and without endotoxin stimulation. Thus, the effects of the peptide fractions on pro-inflammatory cytokine gene expression and protein secretion were comparatively assessed under both standard (basal) and lipopolysaccharide (LPS)-stimulated culture conditions. Ultimately, the context-dependent behavior of these peptides, including their capacity to prime innate immune responses and attenuate inflammatory responses, was elucidated.

## 2. Results

### 2.1. Antioxidant Activities of Crude MBPH and Its Fractions

The antioxidant potential of crude MBPH was comprehensively evaluated using multiple in vitro assays. Analysis of the bioactive components revealed a TPC of 19.42 ± 0.45 µmol GAE/g and a TFC of 0.53 ± 0.06 µmol QE/g. Furthermore, the crude MBPH exhibited strong radical scavenging capacities, recording DPPH and ABTS values of 7.87 ± 0.01 and 6.86 ± 0.10 µmol TE/g, respectively. Its FRAP value was determined to be 1.20 ± 0.01 µmol TE/g. Most notably, its markedly high ORAC value of 311.94 ± 18.27 µg TE/g highlighted the presence of potent chain-breaking antioxidant components capable of mitigating lipid peroxidation.

To further concentrate these bioactive components and elucidate the relationship between peptide molecular weight and antioxidant efficacy, the crude extract was fractionated. Following size-based ultrafiltration, the antioxidant activities of the four peptide fractions (>5, 3–5, 1–3, and <1 kDa) were compared (Table 1). A clear, inverse relationship between molecular weight and antioxidant capacity was consistently observed across all assays. The <1 kDa fraction exhibited the highest radical scavenging activity (e.g., DPPH: 26.12 ± 0.31 µmol TE/g), which was significantly greater than that of the >5 kDa fraction (*p* < 0.05). Similar size-dependent trends were noted for ABTS, FRAP, TPC, and TFC. Notably, the TPC and TFC of the <1 kDa fraction reached 62.95 ± 1.15 µmol GAE/g and 3.57 ± 0.34 µmol QE/g, respectively.

**Figure 1:**
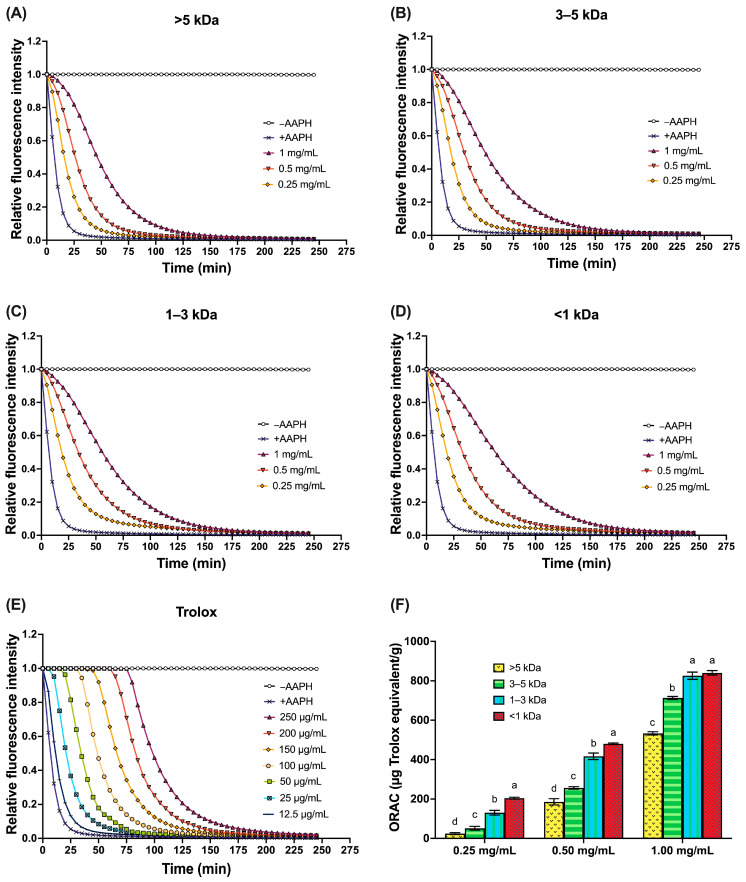
Antioxidant activity of various molecular weight fractions of MBPH (MW > 5 kDa, 3–5 kDa, 1–3 kDa, and <1 kDa), assessed using the oxygen radical absorbance capacity (ORAC) assay. Panels (**A**–**E**) illustrate the decline in fluorescence intensity over time, while Panel (**F**) shows the corresponding ORAC values expressed as μmol Trolox equivalents per gram. Different superscript letters (a–d) within the same sample concentration indicate statistically significant differences (*p* < 0.05).

The peroxyl radical scavenging capacity, measured via the ORAC assay, further corroborated this size–activity relationship. All fractions inhibited peroxyl radical-induced fluorescence decay in a dose-dependent manner (0.25 to 1.00 mg/mL) (Figure 1A–F). At the highest concentration (1.00 mg/mL), the 1–3 kDa and <1 kDa fractions displayed superior ORAC values of 820.69 ± 18.93 and 836.51 ± 11.81 µmol TE/g, respectively, significantly outperforming the larger peptide fractions (*p* < 0.05).

### 2.2. In Vitro Anti-Inflammatory of MBPH Fractions

#### 2.2.1. COX-2 Inhibitory Activity

**Figure 2:**
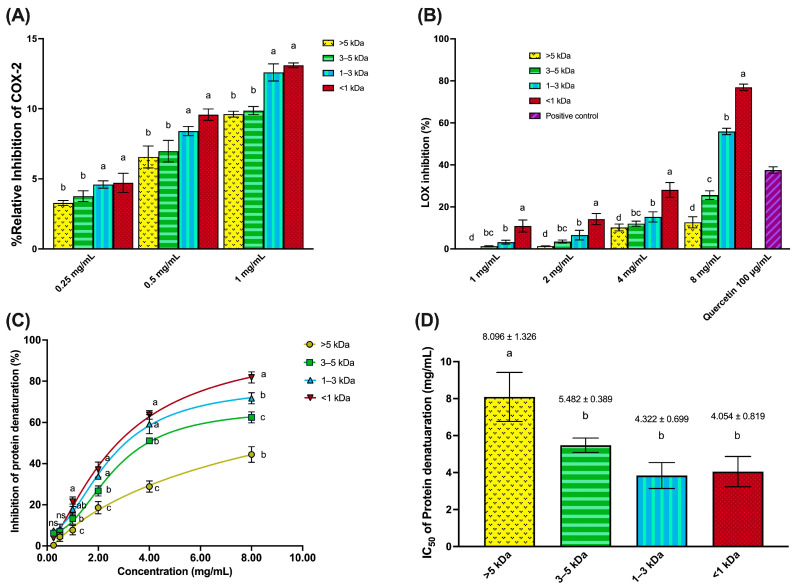
Anti-inflammatory activities of MBPH peptide fractions: (**A**) COX-2 inhibitory activity; (**B**) LOX inhibitory activity; (**C**) inhibition of albumin denaturation; and (**D**) IC_50_ values for albumin denaturation inhibition. Note: Different lowercase letters within the same row at the same concentration indicate statistically significant differences (*p* < 0.05). ns indicates no significant difference (*p* ≥ 0.05).

The COX-2 inhibitory activity of the MBPH fractions was evaluated at concentrations ranging from 0.25 to 1.00 mg/mL (Figure 2A). A clear dose-dependent response was observed, with the LMW fractions consistently outperforming their larger counterparts. At the highest tested concentration (1.00 mg/mL), the <1 kDa and 1–3 kDa fractions exhibited significantly greater inhibition (13.10 ± 0.17% and 12.59 ± 0.61%, respectively) compared to the >5 kDa (9.75 ± 0.21%) and 3–5 kDa (10.00 ± 0.29%) fractions (*p* < 0.05).

#### 2.2.2. LOX Inhibitory Activity

Lipoxygenase (LOX) enzymes play a critical role in metabolizing arachidonic acid into leukotrienes, which are key mediators in chronic inflammatory conditions. To further assess the in vitro anti-inflammatory potential of MBPH, its ability to inhibit LOX was evaluated (Figure 2B). All fractions demonstrated dose-dependent inhibitory activity from 1 to 8 mg/mL, with a strong inverse correlation between molecular weight and LOX inhibition. At 8 mg/mL, the <1 kDa fraction achieved the highest inhibitory activity of 76.93 ± 1.53%, followed by the 1–3 kDa fraction at 55.90 ± 1.53%, whereas the 3–5 kDa and >5 kDa fractions exhibited substantially weaker activities at 25.62 ± 2.04% and 12.67 ± 2.66%, respectively. Additionally, the positive control, quercetin (100 µg/mL), exhibited 37.56 ± 1.55% inhibition.

#### 2.2.3. Albumin Protein Denaturation

Protein denaturation is a well-documented marker for inflammatory diseases, as the loss of secondary and tertiary structures can trigger downstream inflammatory responses. The ability of the MBPH fractions to inhibit heat-induced denaturation of bovine serum albumin was evaluated as an additional measure of anti-inflammatory activity (Figure 2C). All fractions exhibited dose-dependent inhibition across the tested concentrations (0–8 mg/mL). Consistent with the enzyme inhibition assays, the LMW fractions (<1 kDa and 1–3 kDa) exerted a stronger protective effect against thermal denaturation compared to the high-molecular-weight fractions. At 8 mg/mL, the <1 kDa fraction achieved maximum inhibition (81.82 ± 2.72%), followed closely by the 1–3 kDa fraction (71.74 ± 2.69%). This potency was quantitatively confirmed by IC_50_ values (Figure 2D). The <1 kDa and 1–3 kDa fractions displayed the lowest IC_50_ values (4.054 ± 0.819 mg/mL and 4.322 ± 0.699 mg/mL, respectively), indicating the strongest anti-inflammatory activity (*p* < 0.05). Conversely, the >5 kDa fraction was the least potent, with an IC_50_ of 8.096 ± 1.326 mg/mL.

### 2.3. Cytotoxicity

**Figure 3:**
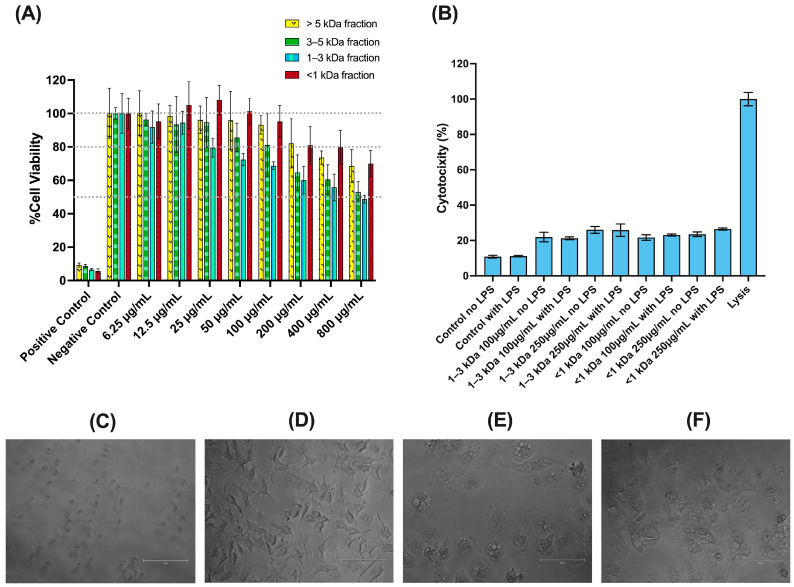
Cytotoxicity of mung bean protein hydrolysate fractions (>5 kDa, 3–5 kDa, 1–3 kDa, and <1 kDa) against RAW 264.7 macrophages following 24 h incubation, determined by (**A**) MTT assay and (**B**) LDH release assay. Values are presented as mean ± SD (*n* = 6). Representative microscopic images of RAW 264.7 macrophages: (**C**) positive control, (**D**) untreated negative control, (**E**) treated with 100 μg/mL 1–3 kDa MBPH fraction, and (**F**) treated with 200 μg/mL 1–3 kDa MBPH fraction. Note: The dotted lines in (**A**) indicate cell viability levels at 50%, 80%, and 100%, respectively, to facilitate easier graph interpretation.

The safety profile of the MBPH fractions (>5, 3–5, 1–3, and <1 kDa) was established in RAW 264.7 macrophages using both MTT and LDH assays to ensure that subsequent immunomodulatory observations were not artifacts of cellular toxicity. As depicted in the MTT assay (Figure 3A), all MBPH fractions demonstrated high biocompatibility at concentrations up to 100 μg/mL, maintaining cell viability above 90%. Interestingly, a slight increase in viability (>100%) was observed at 25–50 μg/mL for the <1 kDa fraction. Conversely, a dose-dependent reduction in viability occurred at higher concentrations (200–800 μg/mL). The IC_50_ values for the 1–3 kDa and 3–5 kDa fractions were 684.76 and 716.70 μg/mL, respectively, whereas the IC_50_ values for the >5 kDa and <1 kDa fractions exceeded 800 μg/mL.

To verify that the observed viability trends were not confounded by membrane damage, LDH release was measured as an indicator of cellular membrane integrity (Figure 3B). Treatment with MBPH fractions resulted in modest baseline cytotoxicity (~20–27%), which was substantially lower than the 100% lysis control. Consistent with the MTT results, cell viability at 100 µg/mL remained sufficiently high (~73–80%). Further evidence was provided by morphological studies as well (Figure 3C–F). Results showed that, unlike the positive control, which exhibited shrinkage and detachment (Figure 3C), cells exposed to MBPH fractions appeared adherent and extended, similar to the negative control (Figure 3D), but with numerous intracellular vacuoles.

### 2.4. Cytokine Gene Expression Profiling

**Figure 4:**
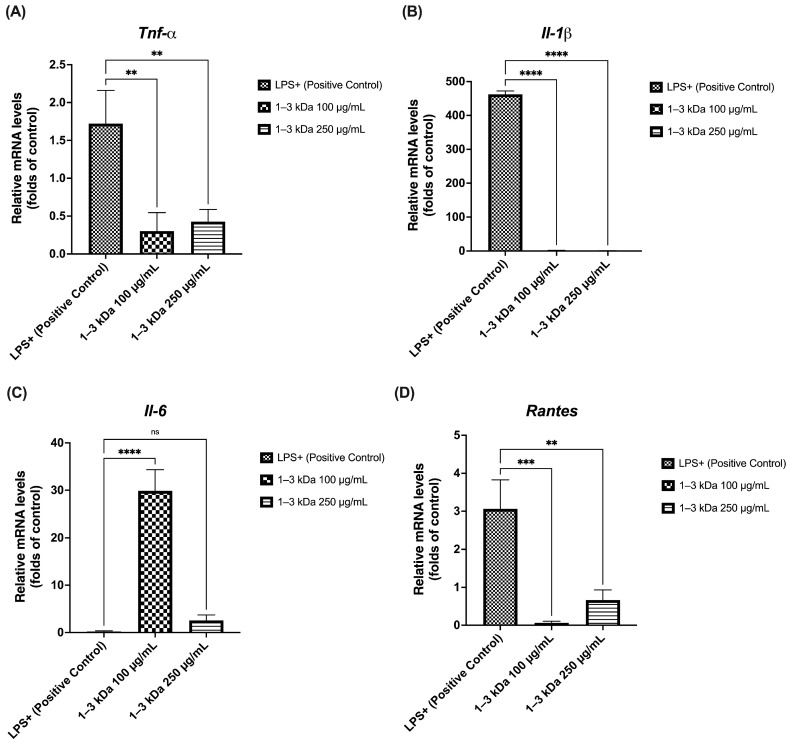
Relative mRNA expression levels of inflammation-related cytokines in RAW 264.7 macrophages after 3 h treatment with MBPH fraction (1–3 kDa): (**A**) *Tnf-α*, (**B**) *Il-1β*, (**C**) *Il-6*, and (**D**) *Rantes*. Note: Gene expression levels are presented as fold changes relative to the basal (LPS−) condition, which was set to 1. The LPS-stimulated group without treatment is represented as the LPS+ (positive control). Data are presented as mean ± standard deviation (SD). Statistical significance is indicated as ** *p* < 0.01, *** *p* < 0.001, and **** *p* < 0.0001 compared with the untreated control; ns, not significant.

To further elucidate the molecular mechanisms underlying the anti-inflammatory and immunomodulatory effects of the 1–3 kDa MBPH fraction, the mRNA expression levels of four key pro-inflammatory mediators were quantified by RT-qPCR following 3 h of incubation. Gene expression in RAW 264.7 macrophages was assessed under two different conditions: basal (LPS−) and inflammatory (LPS+). The 1–3 kDa fraction treatment under (LPS+) inflammatory conditions showed a strong reduction in early-response gene expression for *Tnf-α* and *Il-1β* when compared to the LPS+ (positive control) group (Figure 4A,B). In contrast, Il-6 expression exhibited a distinct pattern (Figure 4C): under basal conditions, the 1–3 kDa fraction produced a marked increase in *Il-6* expression. Treatment showed no effect on *Rantes* expression at this time point (Figure 4D).

### 2.5. ELISA Quantification of Il-6

**Figure 5:**
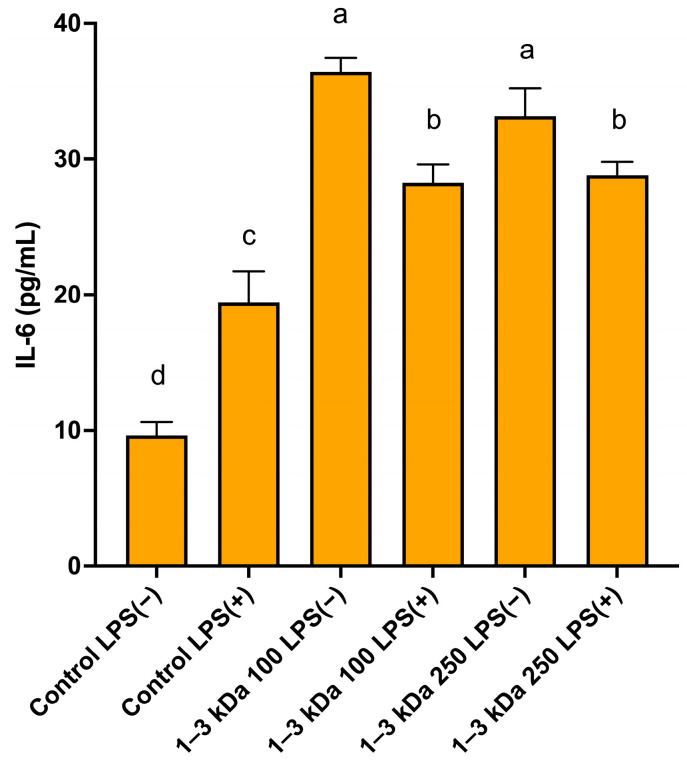
Effects of MBPH fractions on IL-6 secretion from RAW 264.7 macrophages after 6 h of treatment. Note: Values within the same bar graph at the same concentration with different lowercase letters indicate statistically significant differences (*p* < 0.05).

To validate whether the early transcriptional modulation observed at 3 h translates into functional extracellular protein release, IL-6 secretion was quantified by ELISA at 6 h post-incubation (Figure 5). At 100 µg/mL, baseline (LPS−) conditions showed that 1–3 kDa fraction treatment resulted in a 36.42 ± 1.02 pg/mL increase in IL-6 secretion. Under inflammatory (LPS+) conditions, IL-6 secretion was 28.25 ± 1.36 pg/mL, lower than the corresponding basal value at the same concentration. The same tendency appeared at 250 µg/mL. IL-6 secretion under basal (LPS−) conditions reached 33.15 ± 2.08 pg/mL, while levels dropped to 28.81 ± 1.00 pg/mL after LPS stimulation (LPS+).

## 3. Discussion

### 3.1. Antioxidant Activities of Crude MBPH and Its Fractions

The present study demonstrated that the antioxidant activity progressively increased by reducing molecular weight of peptides, with the <1 kDa fraction exhibiting the greatest radical scavenging ability, reducing power, and phenolic compounds. This trend is consistent with previous reports indicating that the ultrafiltration method is effective in concentrating LMW antioxidant peptides in plant protein hydrolysates. Such fractions are generally considered to possess greater accessibility to free radicals and metal ions owing to their smaller molecular size and higher solubility [20].

Similar observations have been reported for MBPH. In the bromelain-derived mung bean meal protein hydrolysate, the <1 kDa fraction had the highest DPPH radical scavenging activity, whereas its ferric reducing antioxidant power was lower than that of the crude hydrolysate. In addition, mung bean meal protein-derived peptides fractionated by reversed-phase chromatography showed that hydrophilic LMW fractions exhibited stronger radical scavenging activity, but more hydrophobic fractions had higher ferric reducing and metal-chelating activities [21]. These findings suggest that different peptide populations may preferentially contribute to distinct antioxidant mechanisms rather than a single dominant pathway.

The inverse relationship between molecular weight and antioxidant activity observed in the present study is further supported by studies on other legume protein hydrolysates. Similarly, chickpea protein hydrolysate fractions obtained by the same four molecular weight ranges (5–10, 3–5, 1–3 and ≤1 kDa) were found to exhibit the highest scavenging activity for ABTS and DPPH radicals among other fractions indicating enhanced antioxidant properties in LMW fractions across legume species [22]. Simultaneously, another study performed on black beans demonstrated the importance of the molecular weight distribution as one of the main factors responsible for the antioxidant activity of protein hydrolysates but with a different optimal range for each protein source [23]. Collectively, these results suggest that enrichment of antioxidant activity in LMW fractions is a common feature of legume protein hydrolysates, although the most active fraction might be different for each source of protein.

Interestingly, the <1 kDa fraction also exhibited the highest ORAC value along with the highest TPC. This finding suggests that both the phenolic constituents and the bioactive peptides may be involved in the chain-breaking antioxidant activity of this fraction. Positive associations between TPC and ORAC activity have also been reported for several cereal and legume protein hydrolysates [24], supporting the potential contribution of phenolic compounds to the enhanced antioxidant activity found in the present study.

In addition, the elevated antioxidant efficacy of the <1 kDa fraction may also reflect the greater molecular mobility of smaller peptides and the increased exposure of radical-quenching amino acid residues, particularly hydrophobic and aromatic residues such as Tyr, Trp, and Phe, following enzymatic hydrolysis [25,26]. These structural characteristics facilitate electron donation and radical stabilization, which are well-recognized determinants of antioxidant peptide activity. Consequently, the <1 kDa MBPH fraction represents a promising source of natural antioxidant ingredients for future functional food and nutraceutical applications.

### 3.2. In Vitro Anti-Inflammatory of MBPH Fractions

#### 3.2.1. COX-2 Inhibitory Activity

Although the absolute inhibition levels were moderate, the significant differences between fractions underscore the functional importance of peptide size. The superior efficacy of the LMW peptides is likely due to their enhanced accessibility to the enzyme’s active site, consistent with recent findings in peanut worm collagen hydrolysate, where the smallest size fraction (<1 kDa) displayed maximum COX-2 inhibitory efficacy, and molecular docking studies showed that these small peptides interact with the COX-2 enzyme via hydrogen bonding and hydrophobic interactions at the COX-2 active site residues [27]. A similar active-site-binding mechanism, involving short peptides forming hydrogen bonds and hydrophobic contacts with COX-2 active-site residues, has also been reported for walnut dreg protein-derived peptides [28]. By inhibiting COX-2, these peptides can attenuate the production of pro-inflammatory mediators, such as prostaglandins, thereby contributing to the mitigation of chronic inflammation [29].

#### 3.2.2. LOX Inhibitory Activity

The enhanced efficacy of the LMW fractions suggests that smaller peptides are more adept at accessing the enzyme’s active site. In line with the above, a recent investigation into the anti-LOX activity of velvet antler blood hydrolysates found that the smaller peptide fractions (below 3 kDa) and especially the lower ones (below 500 Da) displayed significantly higher anti-LOX activity than the larger-sized counterparts [30]. Furthermore, enzymatic hydrolysis likely enriched these fractions with specific hydrophobic amino acid sequences that possess a higher binding affinity for LOX, as is the case with the smaller fractions from other plant protein hydrolysates [31]. The observation that the <1 kDa and 1–3 kDa fractions demonstrated greater maximal inhibition at higher concentrations than the positive control confirms their intrinsic inhibitory properties and highlights their multi-pronged anti-inflammatory potential.

#### 3.2.3. Albumin Protein Denaturation

The protein denaturing protection against heat-induced unfolding in LMW (<1 and 1–3 kDa) MBPH fractions indicates the significance of molecular weight of peptide in anti-inflammatory action. The smaller peptides are assumed to have better access to the unfolded proteins and thus display a larger amount of hydrophobic and aromatic amino acids created through hydrolytic process and therefore become more effective as protein stabilizers [32,33]. The findings obtained by the current study agree with previous studies on protein hydrolysates from other biological sources. For instance, the enzymatic hydrolysis of proteins from *Rhodomonas* sp. showed significant increases in inhibitory effects against albumin denaturation caused by heating compared to crude proteins, which shows that the formation of peptides via hydrolysis increases their ability to stabilize proteins [34]. Similarly, LMW chickpea protein hydrolysate fractions exhibited greater inhibition of protein denaturation in a concentration-dependent manner, further supporting the molecular-weight-dependent enhancement of anti-inflammatory activity [22].

Overall, the findings consistently highlight LMW peptides serve as the primary elements that determine MBPH’s ability to reduce inflammation. The bioactivity of these fractions was higher in multiple experimental tests, which showed that peptide size determines their functional performance. The research demonstrates that enzymatic hydrolysis produces peptide sequences that possess bioactive properties to better interact with inflammation-related targets providing evidence for their use as functional ingredients in inflammation treatment.

### 3.3. Cytotoxicity

The low cytotoxicity observed across all MBPH fractions indicates relatively low cytotoxicity within the investigated concentration range, consistent with [35], who reported RAW 264.7 viability above 90% across 10–200 µg/mL, with significant cytotoxicity only above 400 µg/mL. The slight increase in viability observed at 25–50 μg/mL for the <1 kDa fraction suggests a mild hormetic response, whereby low peptide doses may stimulate mitochondrial activity. A similar proliferative effect was reported by Diao et al. [36] who demonstrated that all fractions of MBPH increased the proliferation of RAW 264.7 compared to the control, with the lowest molecular weight fraction (<1165 Da) having the most significant effect. This supports a size-dependent stimulation of macrophage metabolic activity.

In line with the MTT results, the LDH results demonstrated that the drop in viability at higher concentrations was not due to membrane disruption, because cytotoxicity in all fractions was below the level of lysis control. The intracellular vacuoles observed in treated cells, in the absence of the shrinkage and detachment characteristic of the positive control, likely signify internalization of the hydrolysate or induction of macrophage activity rather than cell membrane damage. This explanation is consistent with the observation of Diao et al. [36], who also observed that MBPH-treated RAW 264.7 cells showed little morphological changes compared with the dramatic dendritic morphology caused by LPS. The formation of intracellular vacuoles is also in line with the formation of phagosomes during macrophage phagocytosis, which involves recognition, internalization and intracellular processing of particles [37], and has also been reported for immunomodulatory peptides from other legume protein hydrolysates [38].

Based on these cytotoxicity assessments, alongside the superior antioxidant and anti-inflammatory potentials demonstrated in preliminary assays, the fraction in the range of 1–3 kDa was selected for subsequent cytokine gene expression and protein secretion analyses. Both concentrations of 100 and 250 µg/mL were chosen for analysis, representing the highest tested doses that preserved cell viability over a biologically relevant concentration range while remaining well below the IC_50_ values of all MBPH fractions. Collectively, these findings confirm that the selected experimental conditions provided acceptable cytocompatibility, allowing subsequent changes in cytokine gene expression and protein secretion to be interpreted as genuine immunomodulatory responses rather than nonspecific effects resulting from cytotoxicity.

### 3.4. Cytokine Gene Expression Profiling

The transcriptional profiles at this time point revealed distinct modulatory patterns dependent on gene-specific expression kinetics. The reduction in *Tnf-α* and *Il-1β* expression is consistent with the strong cellular permeability of LMW peptides, which leads to their rapid internalization and direct TLR4 pathway disruption according to Medzhitov et al. [39]. The process shows initial blockage of natural inflammatory signaling pathways, which includes NF-κB activation blockage and reactive oxygen species (ROS) accumulation reduction [40].

The cytokine displays peak production at three hours because it requires following TNF-α and IL-1β signaling for its complete development. Under normal conditions, the 1–3 kDa fraction produced a major increase in *Il-6* expression. The basal induction process establishes an initial stage of immune-priming, which occurs when the peptides bind to pattern recognition receptors (PRRs) that prepare innate defenses while avoiding complete systemic inflammation [41,42].

The lack of effect on *Rantes* expression is likely attributable to its slower transcriptional kinetics, which require extended activation of secondary signaling loops not yet engaged at this time point. Collectively, these findings indicate that the 1–3 kDa MBPH fraction controls early inflammatory gene expression through the reduction in essential pro-inflammatory mediators (*Tnf-α*, *Il-1β*), which results in different effects on *Il-6*. Further investigation at the protein level is required to confirm the functional implications of these transcriptional changes.

### 3.5. ELISA Quantification of Il-6

The findings demonstrate that immune system modulation by the 1–3 kDa MBPH fraction varies according to specific situational contexts. At 100 µg/mL, the slight increase in IL-6 secretion under basal conditions suggests only a mild immunostimulatory effect, while the reduction observed under LPS stimulation indicates that inflammatory conditions alter the fraction’s functional response. The consistent pattern at 250 µg/mL, where IL-6 secretion remains below the basal level under inflammatory conditions, further supports a concentration independent but context dependent regulatory effect.

This bidirectional regulation suggests that the 1–3 kDa MBPH fraction exhibits distinct functional roles depending on the cellular environment. The oligopeptides activate macrophages in a controlled manner under normal conditions while they decrease cytokine production during inflammatory stimulation according to Diao et al. [35] and Diao et al. [36]. The effects of this treatment establish links between pattern recognition receptor interactions and downstream signaling pathway modulation, which includes NF-κB [43]. Hydrolysates from various plant sources show similar immunostimulatory activities, which include soy and wheat because their small peptides boost macrophage phagocytic activity and cytokine release without causing any toxic effects [44,45]. The molecular size of peptides determines their bioactive effects because researchers found that peptides between 1 and 3 kDa exhibit both stable properties and effective control over inflammatory response mechanisms according to Li et al. [46].

As reported by Kiewiet et al. [47], certain peptides use surface receptors to create their effects while other peptides use transporters like PepT1 or fluid-phase endocytosis to enter cells and then control NF-κB and MAPK cellular pathways. The 1–3 kDa fraction shows strong effectiveness because peptides within this size range contain physicochemical characteristics that help them enter cells and protect against long-term inflammation. Hence, the research results demonstrate that the 1–3 kDa MBPH fraction functions as an immunomodulatory agent, which depends on specific environmental conditions.

It is noteworthy that the concentration range used in the RAW 264.7 macrophage experiments was different from that used in the antioxidant and cell-free anti-inflammatory assays as these experimental systems represent fundamentally different biological endpoints. Biochemical assays measure direct chemical or enzyme inhibitory interactions, hence wide concentration ranges are needed to derive reliable dose–response and IC_50_ values. For the cell-based experiments, we intentionally used non-cytotoxic concentration ranges as determined by the MTT assay. This ensured that the observed changes in cytokine production were due to genuine immunomodulatory activity and not non-specific effects associated with decreased cell viability.

## 4. Materials and Methods

### 4.1. Materials

Mung bean (*Vigna radiata* L.) was sourced from Thanya Farm Co., Ltd. (Nonthaburi, Thailand). The dehulled beans were soaked for 6 h at ambient temperature, then milled with water at a 1:1 ratio using a wet grinder (PHILIPS HR2068, Philips Electronics (Thailand) Ltd., Bangkok, Thailand). The resultant mixture was dried at 50 °C for 24 h, ground into flour, and sieved through a 100-mesh filter [48]. Defatting of the mung bean flour was performed using hexane at a 1:3 (*w*/*v*) ratio, with stirring at 500 rpm for 40 min [49]. The defatted flour was then dried in a fume hood for 24 h to reduce residual hexane content and stored at 4 °C until further use.

### 4.2. Mung Bean Protein Isolate (MBPI) Preparation

MBPI was prepared using a modified method from Liu et al. [48]. Defatted mung bean flour (5% *w*/*v*) was mixed with distilled water and stirred to 40 °C. The pH was adjusted to 9.5 with NaOH and maintained at 40 °C for 1 h. After centrifugation at 4000 rpm for 10 min, the supernatant was collected and the pH adjusted to 4.6 with HCl. The solution was centrifuged again, and the precipitate was collected, dispersed in water, and the pH adjusted to 7.0 with NaOH. The precipitate was frozen at −80 °C for 12 h and lyophilized at −55 °C under vacuum for 48 h. The final MBPI was stored at room temperature for further use.

### 4.3. MBPH Preparation

MBPH was prepared by enzymatic hydrolysis. First, MBPI was dissolved in distilled water (3% *w*/*v*) and hydrolyzed using alcalase (Sigma Chemical Company, St. Louis, MO, USA) under optimized conditions (enzyme concentration and hydrolysis time) from a previous study [50]. The pH was adjusted to 8.5 and maintained with 1 M NaOH, and the reaction was conducted at 55 °C. Enzyme inactivation was achieved by heating the reaction mixture in a 95 °C water bath for 15 min, followed by cooling to room temperature. The mixture was then centrifuged at 10,000× *g* for 20 min at 4 °C to collect the supernatant. The pH was adjusted to 7.0 before freeze-drying, and the resulting powder was stored for future use.

### 4.4. Ultrafiltration

The hydrolysate obtained from the previous step was subjected to ultrafiltration using a molecular weight cut-off (MWCO) membrane of 5 kDa, 3 kDa, and 1 kDa (VivaFlow 200, Sartorius Stedim Biotech GmbH, Göttingen, Germany) to fractionate peptides and remove impurities. MBPH was prepared at 0.5% (*w*/*v*), and the feed solution was processed at 5 mL/min using Amicon^®^ stirred cells (MERCK, Darmstadt, Germany). This procedure yielded four fractions (>5, 3–5, 1–3, and <1 kDa), which were subsequently lyophilized. The fractions were then analyzed for their antioxidant and anti-inflammatory activities.

### 4.5. Total Phenolic Compound and Total Flavonoid Contents Determination

#### 4.5.1. Total Phenolic Content (TPC)

The TPC of the extracts was quantified using the Folin–Ciocalteu method, adapted from Singleton et al. [51]. MPBH was first dissolved in distilled water to obtain a concentration of 1 mg/mL. An aliquot of 20 µL from each sample was transferred into a 96-well plate, followed by the addition of 100 µL of 10% Folin–Ciocalteu reagent. Subsequently, 80 µL of 7.5% (*w*/*v*) sodium carbonate (Na_2_CO_3_) solution was added, thoroughly mixed, and incubated in the dark for 2 h. Absorbance was then recorded at 730 nm using a microplate reader (Spark, Tecan, Hombrechtikon, Switzerland), and the standard calibration curve was constructed using gallic acid. TPC values were calculated based on the standard equation and expressed as milligrams of gallic acid equivalents (mg GAE) per gram of dry weight.

#### 4.5.2. Total Flavonoid Content (TFC)

The TFC was assessed using the aluminum chloride colorimetric method [52]. A 100 µL aliquot of the hydrolysate was mixed with 100 µL of aluminum chloride (10%), 100 µL of potassium acetate (1.0 M), and 2.8 mL of distilled water. The reaction mixture was incubated at room temperature for 30 min, and the absorbance was measured at 415 nm. Results were expressed as mg of quercetin equivalents (QE) per gram of sample.

### 4.6. Antioxidant Activities

#### 4.6.1. DPPH Radical Scavenging Activity Assay (DPPH)

The DPPH free radical scavenging activity was determined using the method described by Tanzadehpanah et al. [53]. A 1 mL aliquot of the hydrolysate was mixed with 2 mL of 0.1 mM DPPH solution. The reaction was incubated for 30 min in the dark, and the absorbance was measured at 517 nm. The results were then compared to the Trolox standard and expressed as micromoles of Trolox equivalent (TE) per gram of sample (µmol TE/g).

#### 4.6.2. ABTS Radical Cation Decolorization Assay (ABTS)

ABTS radical cation decolorization assay was performed according to the method by Re et al. [54]. The ABTS solution was prepared by reacting 7 mM ABTS with 2.45 mM potassium persulfate and incubating the mixture 12–16 h at room temperature. The solution was first adjusted by dilution with distilled water until the absorbance reached 0.700 ± 0.002 at 734 nm. Then, 20 µL of hydrolysate was mixed with 180 µL of ABTS solution, followed by absorbance measurement at 734 nm after a 6 min incubation period. The results were compared with the Trolox standard and expressed as micromoles of Trolox equivalents per gram of sample (µmol TE/g)

#### 4.6.3. Ferric Reducing Antioxidant Power (FRAP)

FRAP assay was carried out as described by [55]. The FRAP reagent was prepared by mixing acetate buffer (pH 3.6), TPTZ solution (10 mM in 40 mM HCl), and ferric chloride (20 mM) in a ratio of 10:1:1. A 10 µL aliquot of the hydrolysate was mixed with 190 µL of FRAP reagent and incubated at 37 °C for 30 min. The absorbance was then measured at 595 nm. FRAP values were expressed as µmol TE/g by comparison with a Trolox calibration curve.

#### 4.6.4. Oxygen Radical Absorbance Capacity (ORAC) Assay

ORAC assay was measured following the method of Yin et al. [56]. The assay was performed using fluorescein as a probe and AAPH as a peroxyl radical generator. A calibration curve was established using Trolox standards (25–400 μM). Samples were first diluted to 0.25–1.00 mg/mL in 75 mM phosphate buffer (PBS, pH 7.4). For analysis, 20 μL of diluted sample was combined with 20 μL PBS (75 mM, pH 7.4) and 20 μL fluorescein disodium (0.8 mM in phosphate buffer) in a 96-well microplate, followed by thorough mixing. Fluorescence reduction was measured at 5 min intervals across 45 cycles using a multi-detection microplate reader (Spark, Tecan, Hombrechtikon, Switzerland), operating at excitation/emission wavelengths of 485/538 nm. Fluorescein control groups included a baseline decay control (−AAPH) and a radical-induced control (+AAPH), both lacking antioxidants. The area under the curve (AUC) and net AUC were computed based on Equations (1) and (2). Finally, ORAC values were expressed as μmol/g Trolox equivalents (μmol TE/g) of the MBPH.

AUC = 5 × (f_0_ + f_1_ + … + f_n−1_ + f_n_) − f_0_ − f_n_ (1)

where f_n_ is the relative fluorescence intensity at the nth time point, and the value 5 refers to the 5 min interval separating each measurement.

Net AUC = AUC_Trolox_ − AUC + AAPH (2)

### 4.7. Anti-Inflammatory Potential

The anti-inflammatory activity of the mung bean protein hydrolysate was assessed by evaluating its inhibitory effects on COX-2, LOX, and albumin denaturation.

#### 4.7.1. Cyclooxygenase-2 (COX-2) Inhibitory Activity

COX-2 inhibitory activity was measured using the COX-2 enzyme assay kit (Abcam, Cambridge, UK, Cat. No. ab283401). The assay was performed according to the manufacturer’s instructions. The hydrolysate was incubated with COX-2 enzyme, and the production of prostaglandin E2 (PGE2) was measured fluorometrically at excitation/emission wavelengths of 535/587 nm. The inhibition of COX-2 activity was calculated as a percentage of the control as shown as Equation (3).

% Relative COX inhibition = [(A_control_ − A_sample_)/(A_control_)] × 100 (3)

#### 4.7.2. Lipoxygenase (LOX) Inhibitory Activity

The LOX inhibitory activity was evaluated by dissolving the sample in DI water to a final concentration of 50 µg/mL. An aliquot of 25 µL of this MPBH solution was combined with 975 µL of LOX enzyme (400 U/mL) prepared in 0.2 M borate buffer at pH 9. Subsequently, 1 mL of linoleic acid substrate (250 µM) was added to initiate the reaction. The absorbance was monitored at 234 nm using a microplate reader after 3 min and 5 min of substrate addition. DI water served as the control, while quercetin was employed as standard inhibitors. The percentage of LOX inhibition was determined using the following Equation (4).

% LOX inhibition = [(A_control_ − A_sample_)/(A_control_)] × 100 (4)

#### 4.7.3. Albumin Protein Denaturation

The inhibition of albumin denaturation was evaluated using the method of Derbel et al. [34]. The hydrolysate was mixed with bovine serum albumin (BSA) and incubated at 37 °C for 15 min. The mixture was then heated at 70 °C for 5 min, and the absorbance was measured at 660 nm using a microplate reader (Spark, Tecan, Hombrechtikon, Switzerland). The percentage inhibition of albumin denaturation was calculated by comparing the sample to the control according to Equation (5).

% Albumin denaturation inhibition = [(A_control_ − A_sample_)/(A_control_)] × 100 (5)

### 4.8. In Vitro Studies of Macrophage Interaction with MBPH Fractions

#### 4.8.1. Cell Viability

Murine macrophage RAW 264.7 cells (TIB-71™) were obtained from the American Type Culture Collection (ATCC, Manassas, VA, USA). The cells were cultured in Dulbecco’s Modified Eagle Medium (DMEM; Gibco, Thermo Fisher Scientific, Grand Island, NY, USA) supplemented with 10% fetal bovine serum (FBS; Gibco, Thermo Fisher Scientific, Grand Island, NY, USA), and incubated at 37 °C in a humidified atmosphere with 5% CO_2_. The culture medium was replaced every 2–3 days. Cell viability was assessed using two methods MTT following the method of Hankittichai et al. [57] and LDH assays.

##### MTT Assay

Cells were seeded into 96-well plates at a density of 1 × 10^5^ cells per well and allowed to adhere 24 h. before exposure to MBPH fractions at varying concentration (0–0.8 mg/mL) for 24 h. Then, MTT solution (3-(4,5-dimethylthiazol-2-yl)-2,5-diphenyltetrazolium bromide) at concentration of 5 mg/mL was added to each well, and the plates were incubated at 37 °C with 5% CO_2_ for 3 h. After incubation, the excess MTT dye solution was removed. The formazan crystals formed were dissolved in dimethyl sulfoxide (DMSO), and the absorbance was measured at 590 nm using microplate reader (Infinite M Plex microplate reader, Tecan, Switzerland). The percentage of cell viability was calculated relative to the control. The concentrations used in the subsequent RAW 264.7 experiments were selected based on the cytotoxicity profile obtained from the MTT assay to minimize nonspecific cytotoxic effects during evaluation of cytokine responses.

##### LDH Assay

Lactate dehydrogenase (LDH) leakage was measured using the Cytotoxicity LDH Assay Kit (MedChemExpress, Monmouth Junction, NJ, USA; Cat. No. HY-K0301). The absorbance at 490 nm was measured to determine the extent of cytotoxicity and cell injury.

#### 4.8.2. Cytokine Gene Expression Profiling

Following treatment, total RNA was extracted from RAW 264.7 macrophages using MagZol Reagent (Magen, Guangzhou, China; Cat. No. R480101). For cDNA synthesis, 1 µg of the isolated total mRNA was subsequently reverse transcribed into cDNA using the iScript Reverse Transcription Supermix (Bio-Rad, Hercules, CA, USA). The primer sequences used for quantitative polymerase chain reaction (qPCR), including those designed for the glyceraldehyde-3-phosphate dehydrogenase (*Gapdh*) reference gene, are provided in Table 2. The analysis was performed using a QIAquant 96 Real-Time PCR System (Qiagen, Hilden, Germany) with cytokine-specific primers. Briefly, the 10 μL qPCR reaction mixture contained 1 μL of cDNA template, 0.375 μL of forward primer, 0.375 μL of reverse primer, 5 μL of SYBR Green Supermix, and 3.25 μL of RNase-free water. The relative gene expression levels of interleukin *(Il)-6*, tumor necrosis factor *(Tnf)-α*, interleukin *(Il)-1β*, and regulated upon activation, normal T cell expressed and secreted (*Rantes*) were determined using the 2^−ΔΔct^ method. All expression levels were normalized to the glyceraldehyde-3-phosphate dehydrogenase (Gapdh) reference gene. The basal condition without LPS stimulation (LPS−) was used as the calibrator, representing a fold change value of 1. The LPS-stimulated macrophages without the peptide fraction treatment were designated as the positive control (LPS+).

#### 4.8.3. Determination of Interleukin-6 (Il-6) Production

RAW 264.7 macrophages were seeded in 24-well plates at a density of 2 × 10^5^ cells per well and allowed to adhere overnight. Cells were then pretreated with MBPH and its peptide fractions at various concentrations (0–0.25 mg/mL) for 6 h and 24 h prior to stimulation with lipopolysaccharide (LPS, 1 ng/mL). Following incubation, culture supernatants were collected for the determination of *Il-6* production. *Il-6* levels were quantified using a commercial enzyme-linked immunosorbent assay (ELISA) kit (FineTest, Wuhan, China; Cat. No. EM0121) according to the manufacturer’s protocol. Absorbance was measured at 450 nm using a microplate reader (Infinite M Plex microplate reader, Tecan, Switzerland), and *Il-6* concentrations were calculated from the corresponding standard curve.

### 4.9. Statistical Analysis

All experiments were independently performed three times. For the antioxidant assays, in vitro anti-inflammatory assays, RT-qPCR, and ELISA, each treatment was analyzed in triplicate. Meanwhile, the MTT and LDH assays were conducted using six technical replicates per treatment. All results are presented as mean ± standard deviation (SD). All statistical analyses were conducted using SPSS statistical software version 17.0 (SPSS Inc., Chicago, IL, USA). Analysis of variance (ANOVA) followed by Duncan’s multiple comparison test was used to determine significant differences between samples (*p* < 0.05).

## 5. Conclusions

The present study demonstrates that the bioactivity of MBPH fractions is strongly influenced by peptide molecular weight, with LMW peptides providing superior bioactive properties. The study found that peptide size and antioxidant capacity showed an inverse relationship that reached its peak in the 1–3 kDa and <1 kDa fractions. The fractions demonstrated strong anti-inflammatory properties through their ability to block COX-2 and LOX pathways and their capacity to prevent albumin protein denaturation. The 1–3 kDa fraction exhibited context-dependent immunomodulatory activity in RAW 264.7 macrophages. Specifically, treatment attenuated early inflammatory responses by suppressing TNF-α and IL-1β production during LPS stimulation, but it resulted in low IL-6 secretion during basal conditions, which suggested immune system priming. The reduction in IL-6 production under inflammatory conditions demonstrated its role as an anti-inflammatory protein. Hence, the research findings demonstrate that LMW MBPH peptides function as both antioxidant and immunomodulatory substances, which can block excessive inflammation while preserving normal immune system activity, highlighting their potential as functional ingredients for health-promoting foods. Nevertheless, the present study was limited to in vitro models. Moreover, because the biochemical and cell-based assays were designed to evaluate different biological endpoints, direct comparison of their effective concentration ranges should be interpreted with caution. Future studies should further purify and identify the bioactive peptide sequences through bioactivity-guided fractionation and validate their efficacy and mechanisms of action by integrating biochemical, cellular, and appropriate in vivo models. Such studies will help establish the concentration–response relationship more comprehensively and further support the development of MBPH-derived functional food ingredients.

## Figures and Tables

**Table 1:** TPC, TFC, and antioxidant activities (DPPH, ABTS, and FRAP) of ultrafiltrated mung bean protein hydrolysate peptide fractions.

Antioxidant Activities	>5 kDa	3–5 kDa	1–3 kDa	<1 kDa
TPC (µmol GAE/g)	46.69 ± 1.10 ^d^	53.93 ± 0.64 ^c^	58.04 ± 2.27 ^b^	62.95 ± 1.15 ^a^
TFC (µmol QE/g)	1.99 ± 0.17 ^d^	2.66 ± 0.28 ^c^	3.15 ± 0.23 ^b^	3.57 ± 0.34 ^a^
DPPH (µmol TE/g)	23.00 ± 0.10 ^d^	23.21 ± 0.22 ^c^	23.93 ± 0.06 ^b^	26.12 ± 0.31 ^a^
ABTS (µmol TE/g)	16.09 ± 0.34 ^b^	18.97 ± 0.31 ^a^	19.36 ± 0.71 ^a^	19.36 ± 0.44 ^a^
FRAP (µmol TE/g)	3.83 ± 0.05 ^d^	4.56 ± 0.33 ^c^	5.05 ± 0.06 ^b^	5.43 ± 0.24 ^a^

Note: Data are presented as mean ± standard deviation. Different lowercase letters within the same row indicate significant differences (*p* < 0.05).

**Table 2:** Forward and reverse sequences of primers used to identify inflammation process markers.

Target	Nucleotide Sequence	Fragment Length
*Il-6* F	5′-AGTTGCCTTCTTGGGACTGA-3′	159 bp
*Il-6* R	5′-TCCACGATTTCCCAGAGAAC-3′	
*Tnf-α* F	5′-AGCCCCCAGTCTGTATCCTT-3′	212 bp
*Tnf-α* R	5′-CTCCCTTTGCAGAACTCAGG-3′	
*Il-1β* F	5′-GCCCATCCTCTGTGACTCAT-3′	230 bp
*Il-1β* R	5′-AGGCCACAGGTATTTTGTCG-3′	
*Rantes* F	5′-CCCTCACCATCATCCTCACT-3′	185 bp
*Rantes* R	5′-CCTTCGAGTGACAAACACGA-3′	
*Gapdh* F	5′-AACTTTGGCATTGTGGAAGG-3′	223 bp
*Gapdh* R	5′-ACACATTGGGGGTAGGAACA-3′	

Source: Neacsu et al. [58].

## Data Availability

The original contributions presented in this study are included in the article. Further inquiries can be directed to the corresponding authors.

## Abbreviations

The following abbreviations are used in this manuscript:

AAPH	2,2′-azobis(2-amidinopropane) dihydrochloride
ABTS	2,2′-azino-bis(3-ethylbenzothiazoline-6-sulfonic acid)
ANOVA	Analysis of variance
ATCC	American Type Culture Collection
AUC	Area under the curve
BSA	Bovine serum albumin
cDNA	Complementary deoxyribonucleic acid
COX-2	Cyclooxygenase-2
DMEM	Dulbecco’s Modified Eagle Medium
DMSO	Dimethyl sulfoxide
DPPH	2,2-diphenyl-1-picrylhydrazyl
ELISA	Enzyme-linked immunosorbent assay
FBS	Fetal bovine serum
FRAP	Ferric reducing antioxidant power
GAE	Gallic acid equivalents
*Gapdh*	Glyceraldehyde-3-phosphate dehydrogenase
IL	Interleukin (e.g., Il-1β, Il-6)
LDH	Lactate dehydrogenase
LMW	Low-molecular-weight
LOX	Lipoxygenase
LPS	Lipopolysaccharide
MAPK	Mitogen-activated protein kinase
MBPH	Mung bean protein hydrolysates
MBPI	Mung bean protein isolate
mRNA	Messenger ribonucleic acid
MTT	3-(4,5-dimethylthiazol-2-yl)-2,5-diphenyltetrazolium bromide
MWCO	Molecular weight cut-off
NCDs	Non-communicable diseases
NF-κB	Nuclear factor kappa B
ORAC	Oxygen radical absorbance capacity
PBS	Phosphate buffer
PGE2	Prostaglandin E2
PRRs	Pattern recognition receptors
QE	Quercetin equivalents
qPCR	Quantitative polymerase chain reaction
*Rantes*	Regulated upon activation, normal T cell expressed and secreted
ROS	Reactive oxygen species
RT-qPCR	Reverse transcription quantitative polymerase chain reaction
SD	Standard deviation
TE	Trolox equivalent
TFC	Total flavonoid content
TLR	Toll-like receptor
TNF	Tumor necrosis factor (e.g., Tnf-α)
TPC	Total phenolic content
TPTZ	2,4,6-tripyridyl-s-triazine
